# A dataset for illuminant- and device- invariant colour barcode decoding with cameras

**DOI:** 10.1016/j.dib.2023.109960

**Published:** 2023-12-15

**Authors:** Michela Lecca, Paola Lecca

**Affiliations:** a Fondazione Bruno Kessler, Digital Industry Center, Technologies of Vision, via Sommarive 18, Trento 38123, Italy; b Faculty of Engineering, Free University of Bozen-Bolzano, NOI Techpark - via A. Volta 13/A, Bolzano 39100, Italy

**Keywords:** Colour images, Colour dependence on light, Device and material, Colour marker detection and decoding

## Abstract

Barcodes are visual representations of data widely used in commerce and administration to compactly codify information about objects, services, and people. Specifically, a barcode is an image composed of parallel lines, with different widths, spacing and sizes. Generally, the lines are dark (usually black) on a bright background (usually white) or vice-versa. Thanks to this representation, barcodes can be detected and decoded in a way robust to changes of light and noise. However, using barcodes with several colours for the lines is quite intriguing because it enables boosting the barcode's data capacity. Colour barcodes still pose a challenge today, even though numerous studies on the topic were conducted between 1990 and 2022. The main issue that needs to be solved is the creation of an optical technology able to decode colour sequences regardless of the ambient light, the acquisition and printing or visualisation device, and the physical support on which the barcode is printed or displayed.

To the best of our knowledge, the studies currently available in literature do not provide the experimental data on which they are based, nor are there online databases that can be used for further studies or for training data analysis procedures based on artificial intelligence techniques.

To fill this gap and push further research in this technology, we built COCO-10, a public dataset of colour barcode images, that would like to become a testbench for the development and testing of colour barcode decoding algorithms, taking into account the colour variability due to the light, to the printer and camera gamuts and to the quality of the paper on which the barcode is printed. COCO-10 contains 5400 images of 150 colour barcodes, each of one printed on two white papers with different density and printers and acquired under six illuminations by three smartphones’ cameras. For each colour barcode image, a mask identifying the region occupied by the barcode is released too. The 150 colour barcodes have been generated by colouring the lines of black & white barcodes with colours randomly selected from a palette of ten colours including both warm and colour hues. The name COCO-10 just refers to the fact that the dataset contains COlor BarCOdes with 10 possible colours for each line. We also provide a set of 300 images created as follows. The 150 COCO-10 colour barcodes were synthetically superimposed on 150 cluttered backgrounds, resulting in 150 images. The first 75 (group 1) were printed on thick paper, the others (group 2) on plain paper. Each group was further subdivided into subsets of 25 images, resulting in 3 subgroups, each of which was captured by 2 smartphones’ cameras under one of the 6 illuminants mentioned above. We also provide masks for these images. These images would like to be a benchmark for testing the accuracy of barcode decoding algorithms, bearing in mind that the performance of these algorithms may be influenced by the accuracy of the previous detection of the barcodes themselves in the background.

The total number of images in COCO-10 is 11700, including the 300 synthetic images of the colour barcodes displayed on white and cluttered background, the 5700 real-world images of the colour barcodes printed on white papers and with cluttered backgrounds and their corresponding 5700 masks.

We finally highlight that COCO-10 can be also used for developing and testing algorithms for gamut and tone mapping, machine colour constancy, and colour correction.

Specifications Table

**Table:** 

Subject	Computer Science, Computer Vision and Pattern Recognition
Specific subject area	The data are designed for developing and/or testing algorithms for illuminant- and device- invariant decoding and detection of colour patterns, precisely colour barcodes.
Data format	The dataset contains 11700 images, including both raw and processed pictures. Specifically, in the dataset, there are 5550 colour images in PPM format, 150 colour images in PNG format, 300 colour images in JPG format and 5700 binary images in PBM format.
Type of data	Images
Data collection	We synthetically generated 150 colour barcodes and we printed them on white papers with different density (80 gr/m2 and 160 gr/m2) by suitable printers ([[1], [2]]). We then acquired each printed image by 3 smartphone cameras (see Table 2) under 6 lights (see Table 3), and specify the position of its barcode by a mask computed by a threshold-based segmentation, followed by manual refinement (if needed). We also synthetically created a set of 150 images, each containing a colour barcode over a cluttered background, we printed each of them on paper with density either 80 gr/m2 or 160 gr/m2, and we acquired it by 2 smartphone cameras (see Tables 2 and 9) under one of the 6 lights in Table 3.
Data source location	Institution: Fondazione Bruno Kessler, Digital Industry Center, Technologies of Vision City/Town/Region: Trento Country: Italy GPS coordinates for collected samples/data: 46.068090626782, 11.151054519014957
Data accessibility	Repository name: COCO-10 Direct URL to data: https://zenodo.org/records/10065746 Instructions for accessing the data: Dataset is published under the Creative Commons Attribution - NonCommercial - ShareAlike 3.0

## 1 Value of the Data

• The barcodes currently in use are generally in black and white, since this feature allow a decoding robust to changes of light and noise [[3], [4], [5], [6], [7]]. To the best of our knowledge, although colour barcode technologies have been investigated in the past [8], there are no colour barcode datasets. Our proposed COCO-10 dataset attempts to address this shortcoming by offering a collection of real-world colour barcodes captured by many cameras in varying lighting situations and printed on two types of paper. The dataset presents a variety of difficulties (listed in the following three bullets) crucial for detection and decoding methodologies.
• First, the dataset presents several *colour distortions*, due to the printing process (e.g., printer's gamut, ink physical features), by the paper quality (e.g., paper absorption degree and reflectance), by the acquisition devices (e.g., cameras’ sensor sensitivity and gamut, automatic white balance).
• Second, the dataset presents *geometric distortions*, like changes of scales, in-plane rotation, skew, due to the cameras’ lens, resolution, and field of view and to the mutual position of cameras and printed barcodes. We highlight that the printed papers were manually positioned in front of the camera with the recommendation of putting the paper parallel to the camera as much as possible. Nevertheless, due to the manual action, sometimes perspective distortions occur. *Noise* due to paper slight wrinkles, physical characteristics of the acquisition devices and – in few cases – some *focus blur* due to errors in acquisition are sometimes present too.
• Third, palette colours and barcode structure make barcode decoding particularly challenging. In fact, the COCO-10 palette contains 10 colours, among which some are clearly distinguishable from each other and some that are less so, especially under certain intensities and types of light and for certain acquisition devices. Moreover, unlike in [[5], [9], [10]], in COCO-10 there are no constraints on barcode length and barcode colour repetition are allowed.
• Given the challenges described above, it can be understood how difficult the colour barcode decoding is, for example for clustering-based algorithms (e.g., [5]), and how necessary disposing of testbed datasets for decoding algorithms in real world scenarios is. COCO-10 has also the merit of having been obtained with an acquisition technique within the reach of any user with a camera: this favours the reproducibility and extension of the dataset.
• Finally, the quantity of images provided, and the availability of masks make COCO-10 an ideal resource for machine learning algorithms. Moreover, development and test of algorithms for gamut and tone mapping, machine colour constancy, colour correction are additional tasks that can benefit from the use of this dataset.

## 2 Data Description

**Fig. 1:**
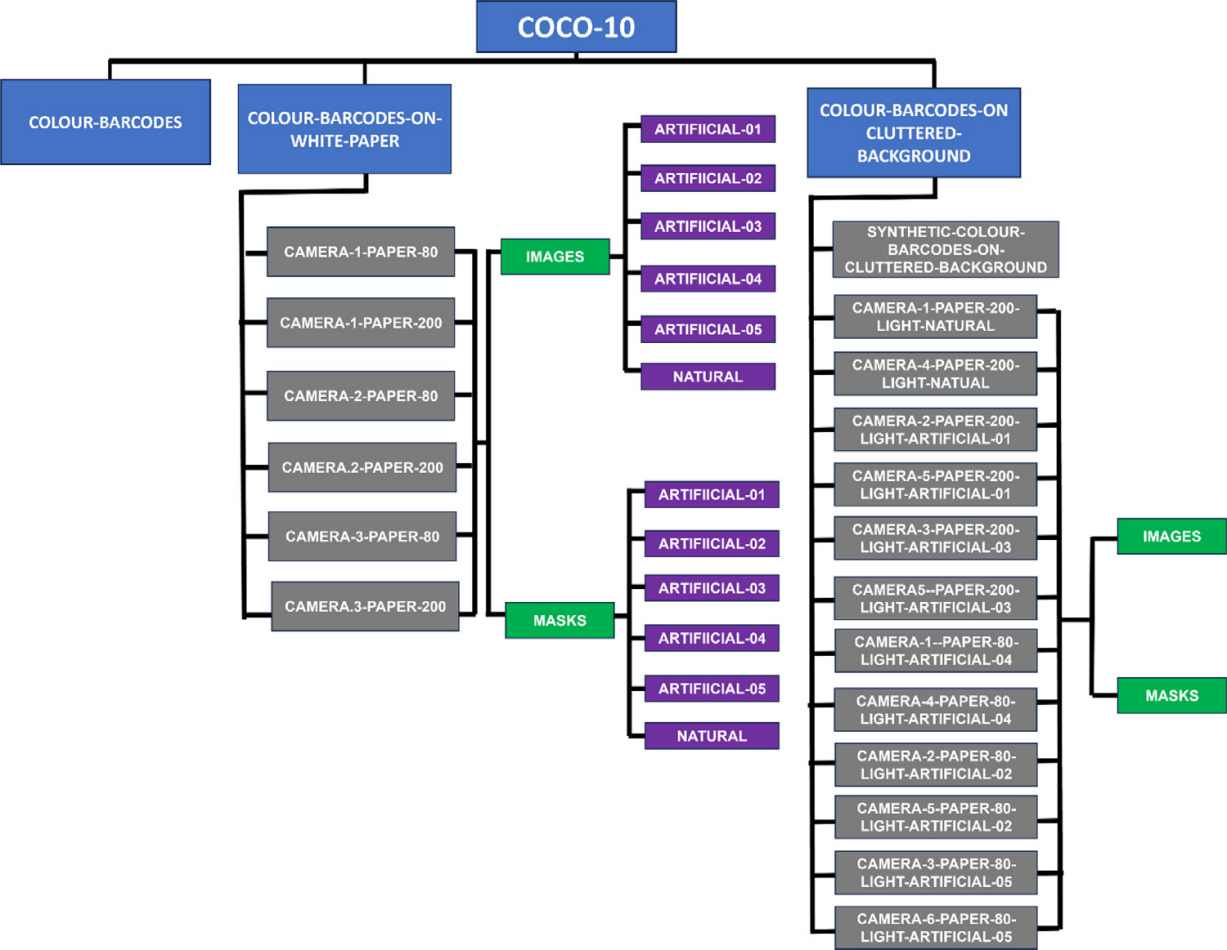
COCO-10 structure. The dataset contains 11400 images and is organized in three main folders, named COLOUR-BARCODES, COLOUR-BARCODES-ON-WHITE-PAPER, and COLOUR-BARCODES-ON-CLUTTERED-BACKGROUND.

**Fig. 2:**
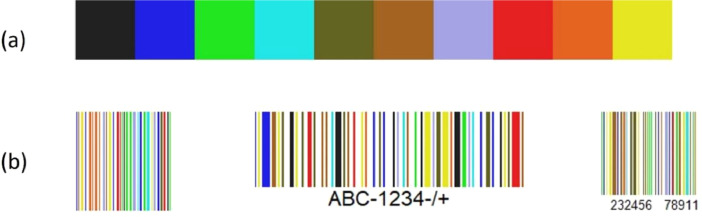
(a) The colour palette of COCO-10; (b) three examples of colour barcodes from COCO-10.
(2) COLOUR-BARCODES-ON-WHITE-PAPER - This directory contains six folders, each of which named CAMERA-X-PAPER-Y, where X indicates the camera used in the acquisition and Y is the density of the white paper on which barcodes have been printed. For instance, directory CAMERA-1-PAPER-80 contains the images of the 150 barcodes printed on white paper with density 80gr/m2 and acquired by Camera 1 under the six illuminations. In each directory, the acquisitions are broken down by the used illumination and stored in subdirectories, called Artificial-01, Artificial-02, Artificial-03, Artificial-04, Artificial-05 and Natural upon the used light. In turn, each of these subdirectories contains the folders images (with the 150 pictures, in PPM format, of the printed colour barcodes) and masks (with the corresponding 150 masks, in PBM format, specifying the region containing the barcode). Images are named 001.ppm, …, 150.ppm and the corresponding masks are named 001.pbm, …, 150.pbm (see Fig. 3, Fig. 4 for some examples).

The dataset COCO-10 contains 11700 images, organized in three main directories (see Fig. 1 and Table 1). Precisely:

(1) COLOUR-BARCODES - This directory includes the 150 images of the colour barcodes synthetically generated form black & white barcodes (see Fig. 2). These images are saved in PPM format and named bc01.ppm, …, bc150.ppm.

**Fig. 3:**
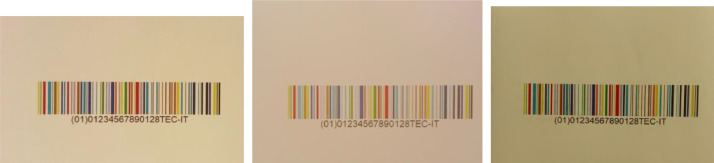
Two images of a colour barcode, both acquired by Camera 1, printed on paper with density 80 gr/m^2^ (on left) and 160 gr/m^2^ (middle). On right, the image of the same barcode printed on paper with density 80 gr/m^2^ but acquired by Camera 6. All these three images have been captured under the illumination Artificial-01. The colours of the left image appear different from those of the middle image because of the different kind of paper, printer, and ink as well as because of the automatic white balance performed by the used camera. The colours of the left image also differ from those of the right image because different cameras render colours differently.

**Fig. 4:**
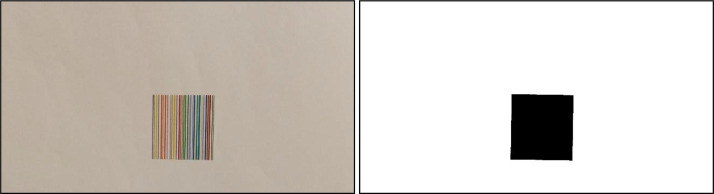
On Left: An image with a colour barcode printed on paper with 80 gr/m^2^ and acquired by Camera 1 under illuminant Artificial-01. On right: The mask of the image on left, i.e., the binary image whose black pixels specify the position of the colour barcode in the image. This mask has been computed by a semi-supervised segmentation algorithm, whose results were eventually refined manually – if needed. A black border has been added to the images to better show the mask content.
(3) COLOUR-BARCODES-ON-CLUTTERED-BACKGROUND - This directory contains 15 folders, specifically:
  (a) directory SYNTHETIC-COLOUR-BARCODES-ON-CLUTTERED-BACKGROUND contains 150 colour images, where the i-th image displays the i-th colour barcode from COLOUR-BARCODES on a cluttered, real-world background; these images are stored in PNG format and named im001.png, … im100.png (see Fig. 5). Backgrounds show indoor or outdoor environments, texture and uniform surfaces, different kinds of objects, like tissues, dolls, food, plants.

**Fig. 5:**
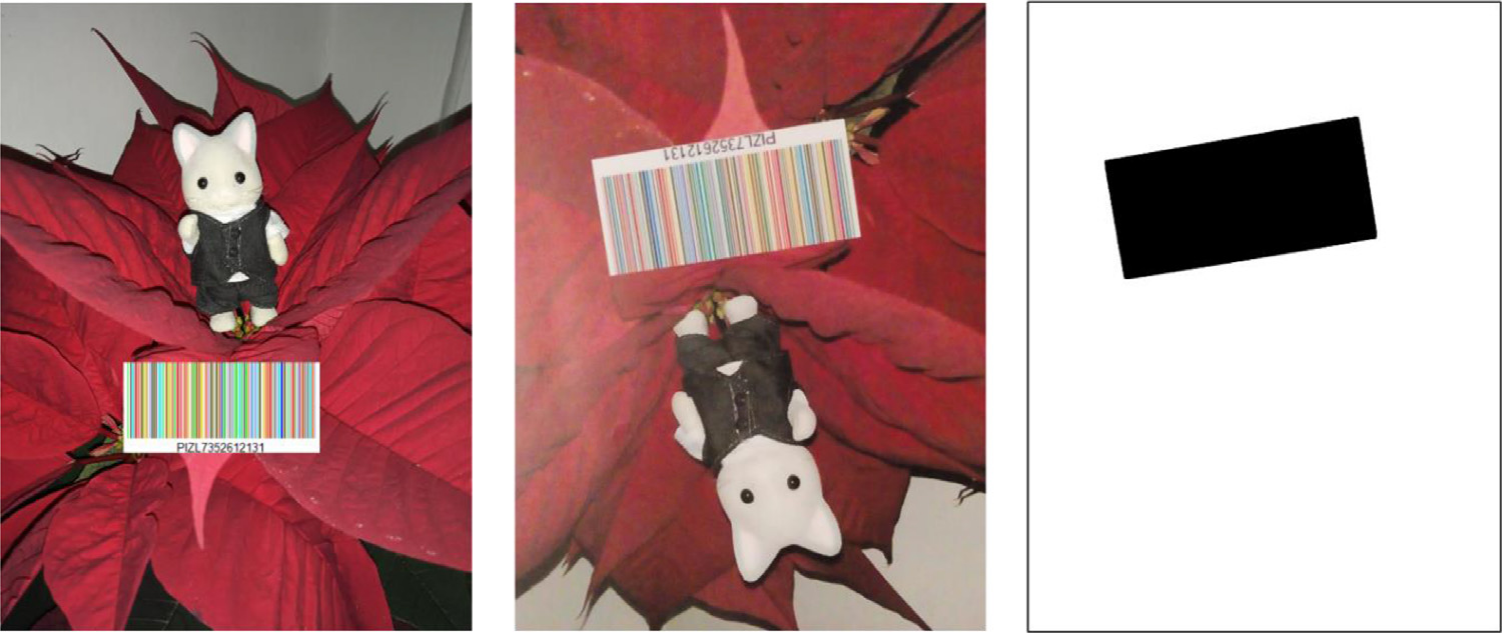
On left: an image from SYNTHETIC-BARCODES-ON-CLUTTERED-BACKGROUND, where a colour barcode appears over a cluttered background; in the midlle: the image on left, printed on a paper with density 80 gr/m^2^, acquired by Camera 6 under illumination Artificial-05; on right, the mask of the image in the middle, where a black contour has been added around the image to better visualize its content.
  (b) 14 folders, each of which is named CAMERA-X-PAPER-Y-LIGHT-Z and contains 25 images from SYNTHETIC-COLOUR-BARCODES-ON-CLUTTERED-BACKGROUND (in JPG format) along with their masks (in PBM format). Precisely, the folder name has the following meaning: X indicates the camera used for the acquisition (X = 1, 2, 3, 4, 5, 6), Y is the density of the paper on which the image has been printed (Y = 80,160 (gr/m^2^)) and Z is the light used in that acquisition (Z = Artificial-01, Artificial-02, Artificial-03, Artificial-04, Artificial-05, Natural). Each of these folders contains in turn two subdirectories named respectively images and masks: the first one contains 25 colour images (named image001.jpg, … image025.jpg) where barcodes are displayed over a cluttered background, while the second one contains the corresponding masks (named masks001.jpg, … masks025.jpg), specifying the colour image region occupied by the barcode. An example is shown in Fig. 5.

**Table 1:** Number of files (i.e., images) and size of the three main directories of COCO-10.

Directory	Number of Files	Size
COLOUR-BARCODES	150	7.55 Mb
COLOUR-BARCODES-ON-WHITE-PAPER	10800	47.60 Gb
COLOUR-BARCODES-ON-CLUTTER-BACKGROUND	750	1.82 Mb

## 3 Experimental Design, Materials and Methods

In this Section we report the steps made for the generation / acquisition of the images in the three main directories of COCO-10, within some analysis performed on the acquired data to provide information about the size of the acquired barcodes and the illuminations.

### 3.1 Images in COLOUR-BARCODES

The first step for the creation of COCO-10 was the generation of its colour barcodes. For this task, we created 150 black & white barcodes from free online websites, i.e. [[11], [12], [13]], and we wrote a specific algorithm for colouring their lines. This algorithm assigns to each line a colour, randomly picked up from the palette of 10 colours shown in Fig. 2(a). The 150 colour barcodes are saved as PPM images and stored in the folder named COLOUR-BARCODES. Some examples are shown in Fig. 2 (b).

Microsoft's pioneer work [5] proposed a colour barcode technology exploiting four colours only and constrained each barcode to contain all these four colours, that were sharply distinguishable from each other, and thus easy to be recognized under several lighting conditions. Although the capacity of these barcodes is quite low, this Microsoft technology was appealing, and other approaches were developed to extend the number of colours. A very good result was obtained in [[9], [10]], where barcodes may contain 24 colours, but their recognition requires several *a priori* information about the sensor gamut and the possible variability range of the illuminations. The palette used in COCO-10 contains 10 colours (see Fig. 2(a)), a number we chose a intermediate point between the 4 colours initially used in [5] and the 24 ones used in [9] and [10]. Our palette contains both warm and cold colours, some of them are clearly distinguishable from each other (see e.g., the first and the last colours in Fig. 2(a)), while others are closer to each other and may become very similar under certain illumination conditions and/or for certain devices (see for instance the fourth and the seventh colours in Fig. 2(a)). These characteristics make COCO-10 very challenging.

### 3.2 Images in COLOUR-BARCODES-ON-WHITE-PAPER

The acquisition of the images stored in COLOUR-BARCODES-ON-WHITE-PAPER consists of three steps, described in the following Subsections.

#### 3.2.1 Colour Barcode Printing on White Paper

The second step of COCO-10 creation was printing the colour barcodes. To this purpose, we considered two kinds of white papers with different densities (80 gr/m^2^ and 160 gr/m^2^ respectively), we arranged two colour barcodes per sheet, and we printed the resulting 75 pages. Given the different paper densities, we used two different printers, i.e. [1] for the paper with the lowest density and [2] for that with the highest density.

#### 3.2.2 Acquisition and Processing of the Barcodes Printed on White Paper

We acquired the colour barcodes printed on the white papers under six illuminations by three smartphone cameras, hereafter denoted as Camera 1, Camera 2 and Camera 3 (see Table 2). These cameras were always used with automatic mode ON, in particular, they always performed automatic white balance.

**Table 2:** Summary of the devices used for acquiring the colour barcodes printed on white paper.

Label	Mark	Resolution
Camera 1	LG K42 - selfie camera [14]	3264×2448
Camera 2	LG K42 - rear camera [14]	4100×3120
Camera 3	realme C11 - rear camera [15]	2448×3264

The illuminations involved in this work are labelled as Natural, Artificial-01, Artificial-02, Artificial-03, Artificial-04 and Artificial-05. The sources, within their correlated colour temperature (where known), are listed in Table 3. They are a natural daylight, four LED lamps, and one halogen lamp. Illuminants Artificial-01, Artificial-04 and Artificial-05 are warm lights, Artificial-04 is a cold light, while Artificial-04 simulates natural, white sunlight. Illuminant Natural is the sun light: the images were collected at different day times and with variable weather conditions, so that it impossible to specify a correlated colour temperature for this illumination.

**Table 3:** Summary of the illuminants used for the acquisition of COCO-10.

Label	Source	Correlated Colour Temperature
Natural	Natural daylight	NA
Artificial-01	Halogen	2800 K
Artificial-02	LED	4000 K
Artificial-03	LED	6400 K
Artificial-04	LED	2800 K
Artificial-05	LED	3000 K

**Fig. 6:**
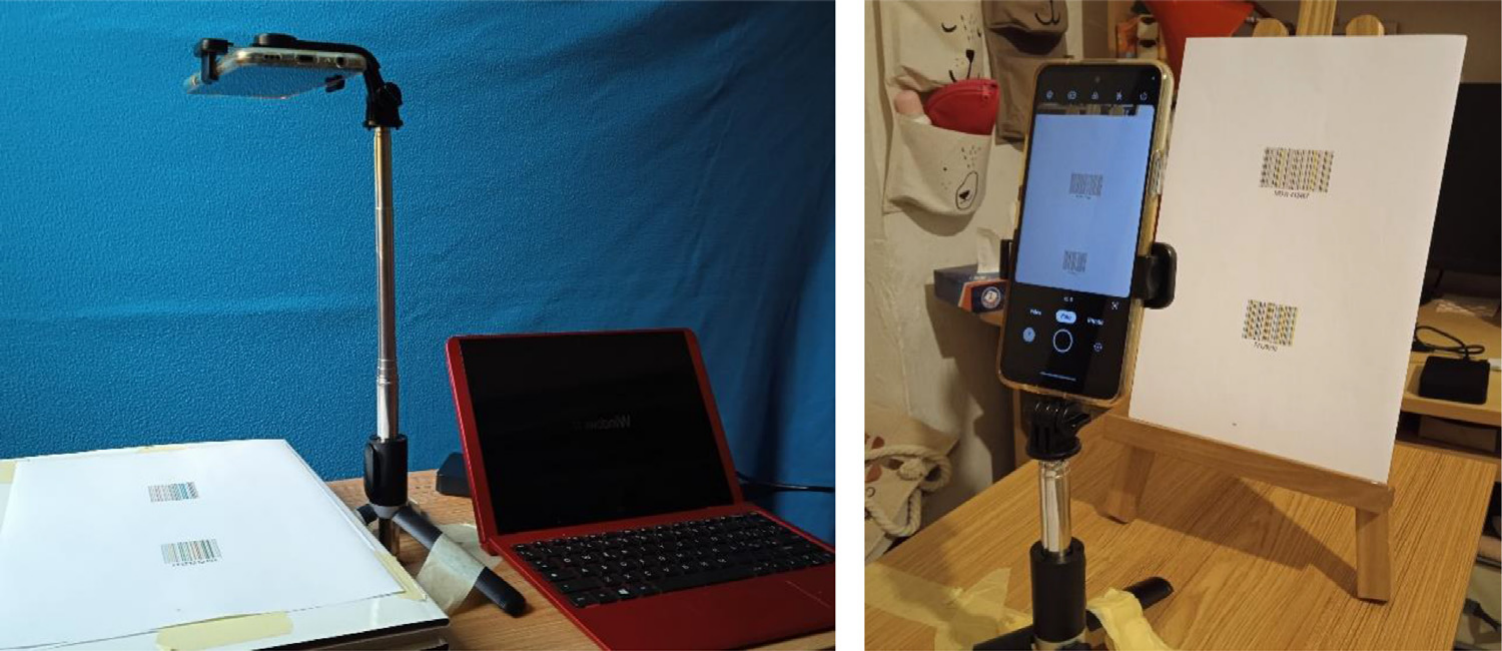
Experimental settings used for the acquisitions of COCO-10. We use the configuration on left or right to minimize shadows caused by the position of the light sources with respect to the camera and to the barcode sheet.

For each camera and for each light, the printed barcodes were acquired as follows. Each paper, depicting two barcodes, was manually located in front of the camera, with the prescription of avoiding (as much as possible) geometric distortions, like skew, perspective changes and remarkable in-plane rotations. To this end, the paper was fixed on a rigid support and the camera tripod was manually adjusted to orient the camera parallel to the paper support (see Fig. 6). Masking tape was used to keep the set up (camera and paper) as immobile as possible to minimise blurring. Nevertheless, in some images we observed some slight geometric distortions due to the manual positioning of the paper. However, the presence of these geometric distortions is not a negative aspect since it adds realism to the data and may be easily corrected by post-processing. We also notify that the size of the barcodes may change from camera to camera due to the different camera physical characteristics (e.g., lens, field of view, resolution) as well as to changes of tripod position from an acquisition to another. Thus, there is no one-to-one correspondence between the images of the same barcode sheets acquired by different cameras and/or by different illuminations.

The 75 images have been then processed and analysed as explained in the following.

#### 3.2.3 Cropping and Masking Colour Barcodes

**Fig. 7:**
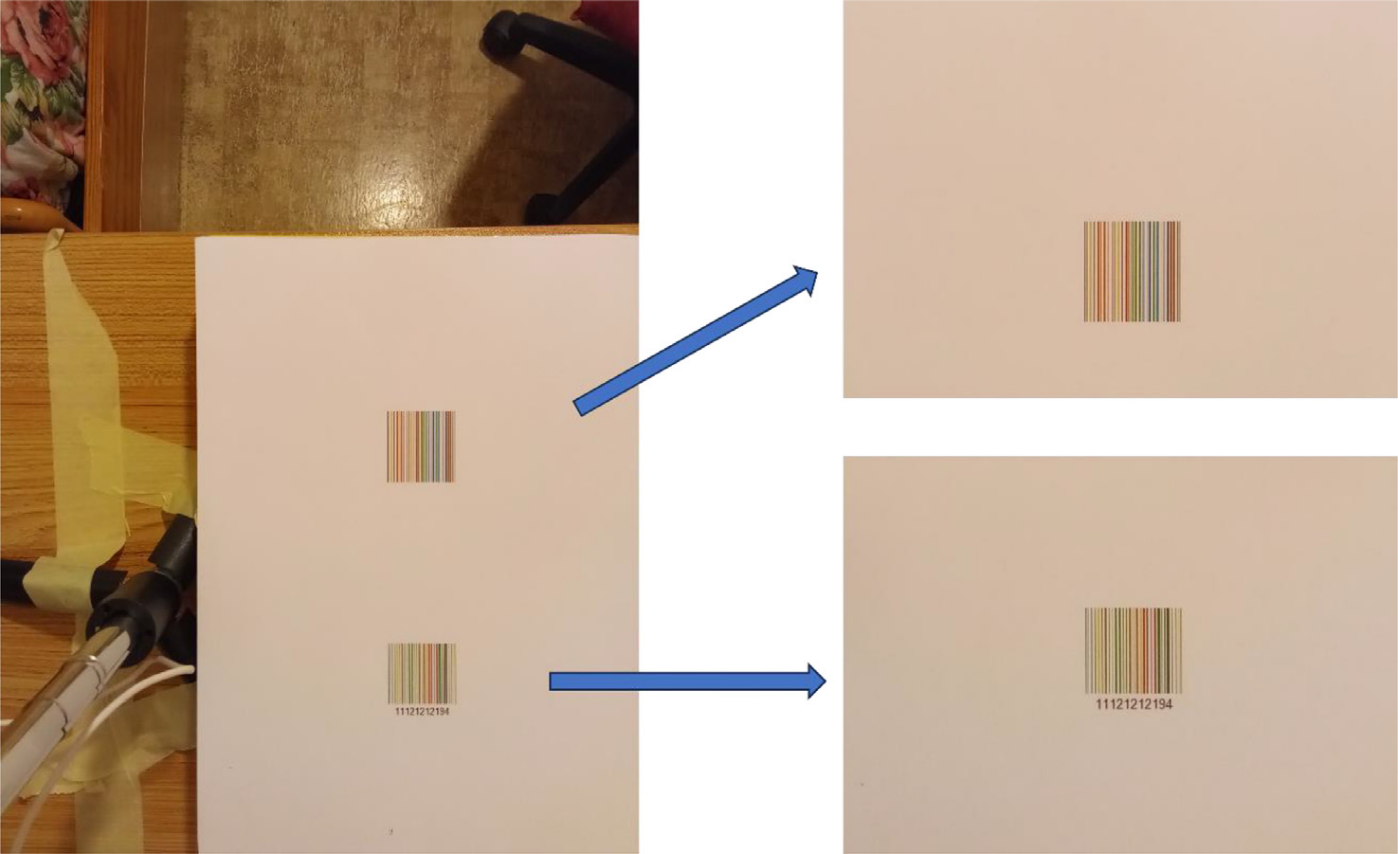
On left, the image of a sheet containing two barcodes acquired by Camera 1 under Artificial-04, and on right the two regions with barcodes cropped out.

Each photo was processed by clipping out two sections that contained barcodes (see Fig. 7). In this way, image parts containing the background or the masking tapes that were used to secure the camera and/or the paper to its support were removed, while we left a portion of the paper around the barcode intact to provide data on how the various cameras under the six illuminations perceived the colour of the same, nearly uniform region (i.e., the white paper). To describe how the cameras “see” a white patch and to analyse colour differences on a uniform area, this information may be helpful.

At the end of this operation, we had 150 colour photographs that we saved as PPM files in the subfolder of COLOUR-BARCODES-ON-WHITE-PAPER relative to the camera and paper used for the acquisition. Then, for each of these photos, we created a mask, which is a binary image in which black pixels correspond to the region occupied by the barcode and white pixels correspond to the white paper surrounding the barcode. To do this, we transformed each colour image to a grayscale image, and we applied to it a semi-supervised threshold-based segmentation. Specifically, this segmentation algorithm takes as input a grayscale image I and creates a binary image B with the same size of I. The values of B are set as follows. The pixels of B whose brightness in I exceeds a certain threshold T are set to 255, while the pixels of the minimum bounding rectangle of the remaining pixels (corresponding in I to the barcode lines) are set to zero. Possible spurious pixels, like very small regions or isolated pixels, are removed by morphological operators. T was set to 90% of the brightness of the 200×200 top square of the input image, which always display a part of white paper. The results were visually examined and – if needed - manually enhanced.

##### 3.2.3.1 Measuring intensity and Chromaticity Changes around Barcodes

We observe that the sub-images with barcodes, cropped out from each sheet image, generally have a different brightness. Specifically, the top sub-image generally is brighter than the bottom one, indicating that the light intensity was varying on the image. This vertical gradient of the light was due to the position of the light with respect to the acquisition plane. In fact, the light source was fixed on the ceiling, about 2 metres from the acquisition desk, but it was impossible to locate it perpendicularly to this desk because of the formation of strong shadows of camera and tripod on the sheet. A horizontal gradient (i.e., an intensity changes from left to right in each sub-image) is also present in some acquisitions, but is in general negligible, especially for the artificial lights. Anyway, the variations of the light intensity and chromaticity on the barcode region are slight, so that each barcode can be considered uniformly illuminated. This claim is supported by the following analysis.

**Fig. 8:**
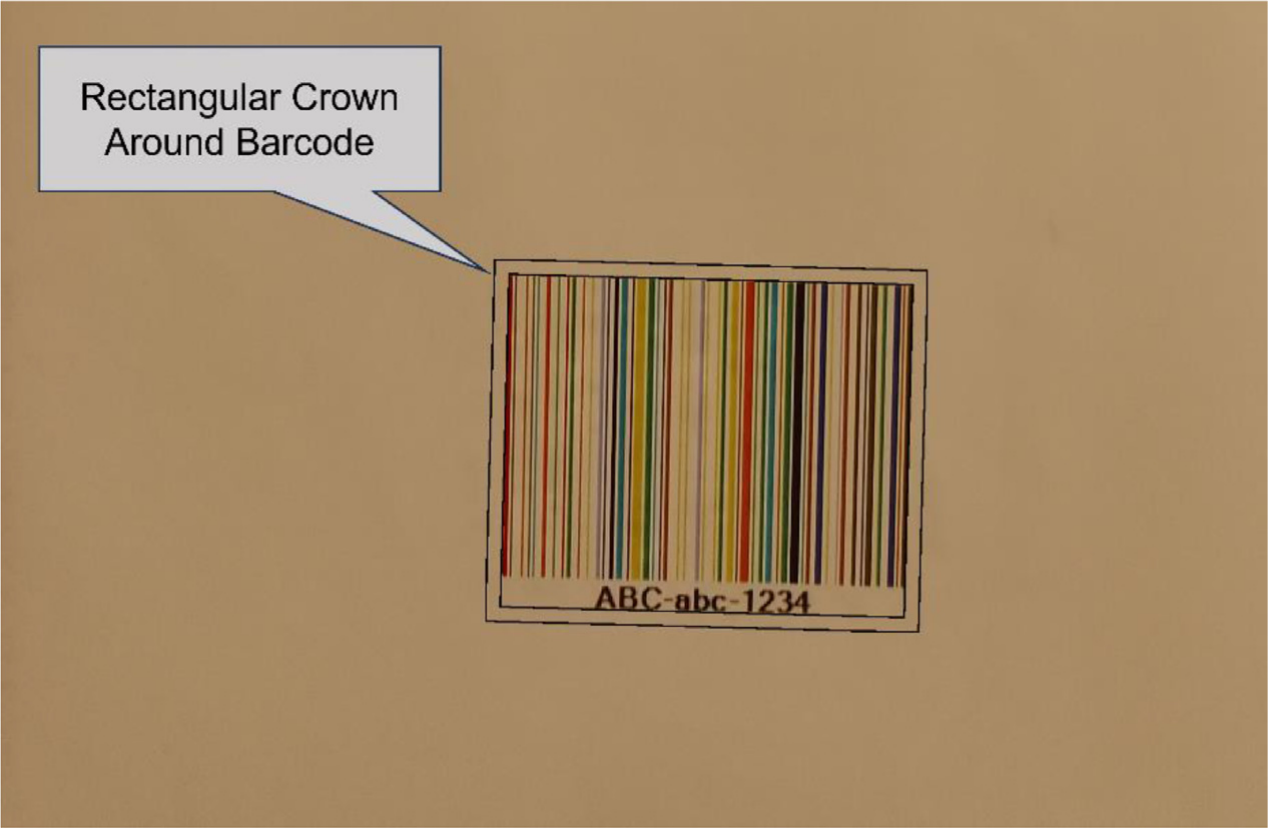
This picture shows the rectangular crown around a colour barcode, printed on white paper with 80gr/m^2^ and acquired by Camera 3 under illuminant Artificial-04. The rectangular crown is used to detect possible variations of intensity and chromaticity of the light near to the barcode.

For any light and for any image of COLOUR-BARCODES-ON-WHITE-PAPER acquired by one of three acquisition cameras, we selected a rectangular crown 20 pixels thick around each barcode (see Fig. 8 for an example) and we computed the mean values and standard deviations of the intensity U and chromaticity (r,g) of their pixels. Mathematically, for any pixel x of the crown, U(x) and (r(x),g(x)) at x are given by:

$$U\left(x\right)=\frac{R\left(x\right)+G\left(x\right)+B\left(x\right)}{3}$$

$$\left(r\left(x\right),\;g\left(x\right)\right)=\left(\;\frac{R\left(x\right)}{R\left(x\right)+G\left(x\right)+B\left(x\right)},\;\frac{G\left(x\right)}{R\left(x\right)+G\left(x\right)+B\left(x\right)}\right)$$

**Fig. 9:**
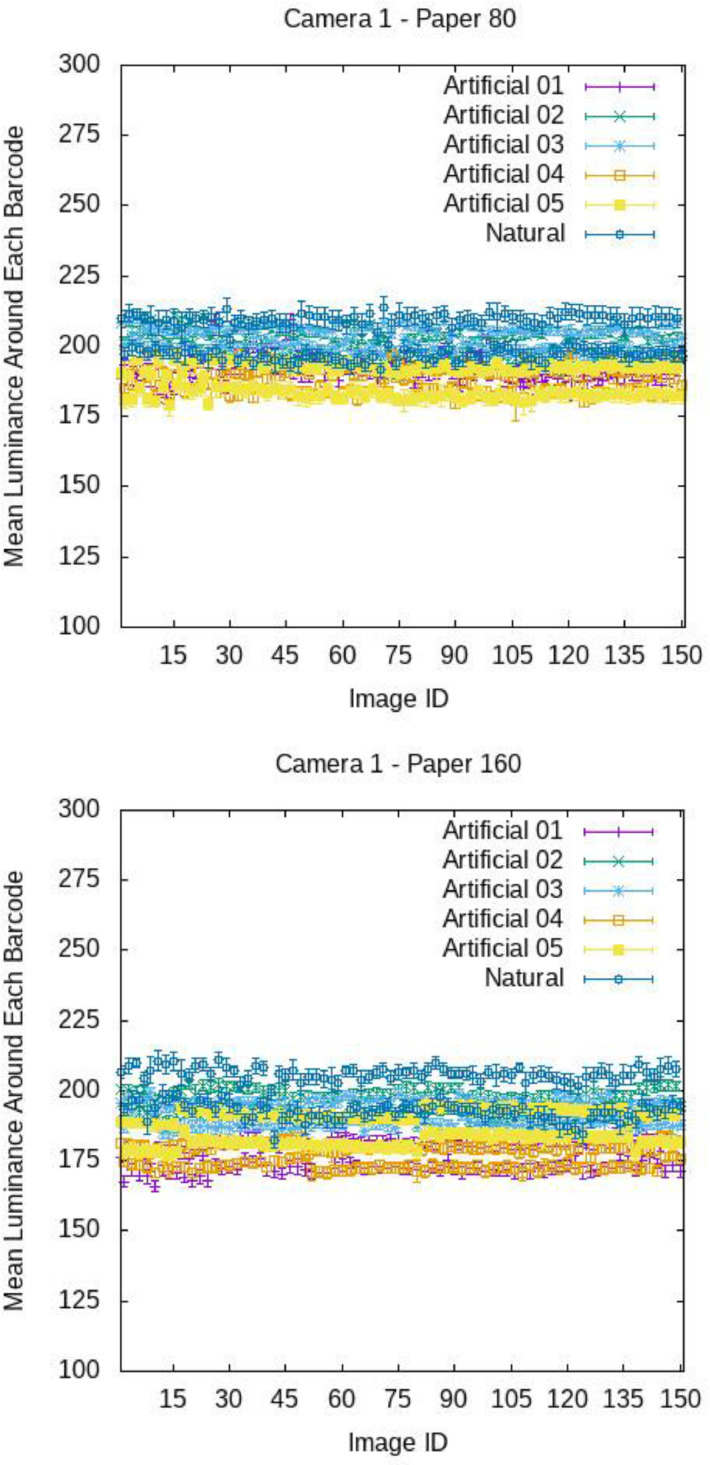
Mean luminance around each barcode for the various images of COLOUR-BARCODES-ON-WHITE-PAPER for Camera 1 and paper with density 80 gr/m^2^ (on top) and 160 gr/m^2^ (on bottom). X-axis reports the label of each image (i.e., 15 refers to the file 015.ppm).

**Fig. 10:**
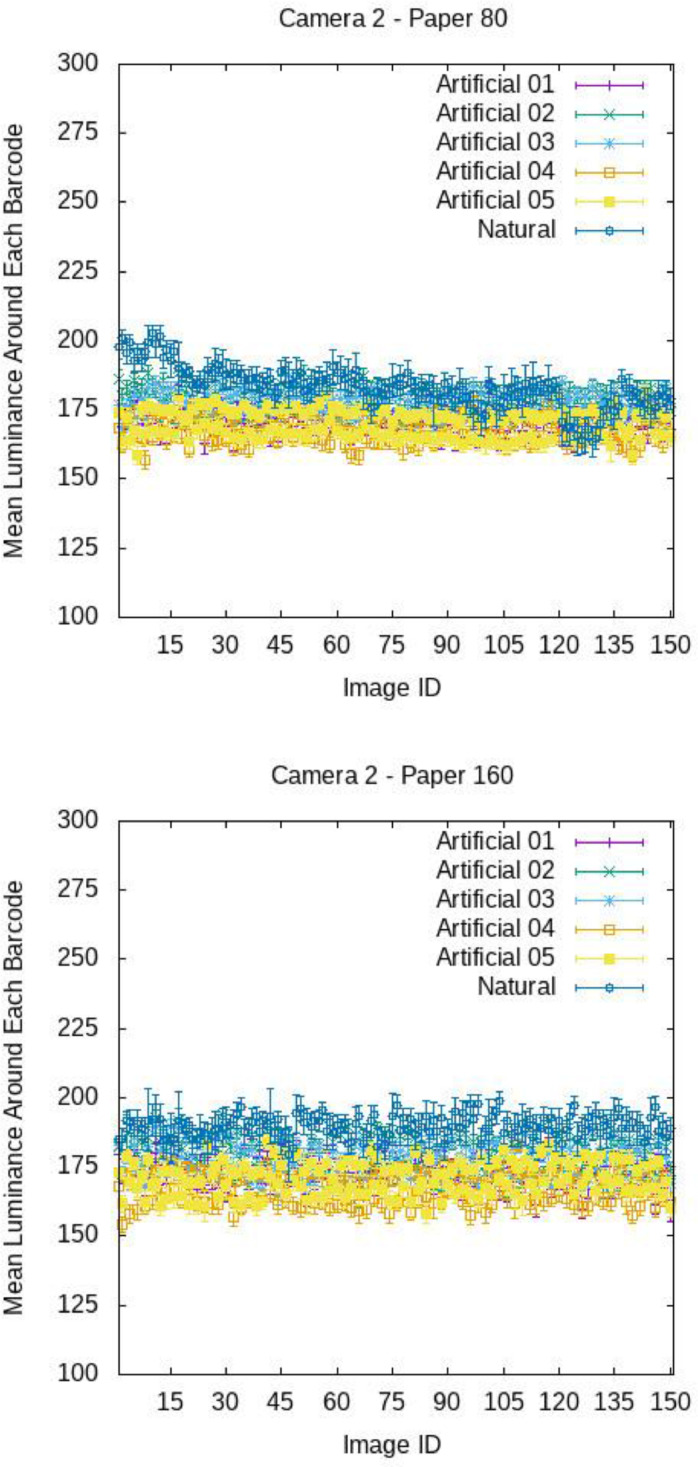
Mean luminance around each barcode for the various images of COLOUR-BARCODES-ON-WHITE-PAPER for Camera 2 and paper with density 80 gr/m^2^ (on top) and 160 gr/m^2^ (on bottom). X-axis reports the label of each image (i.e., 15 refers to the file 015.ppm).

**Fig. 11:**
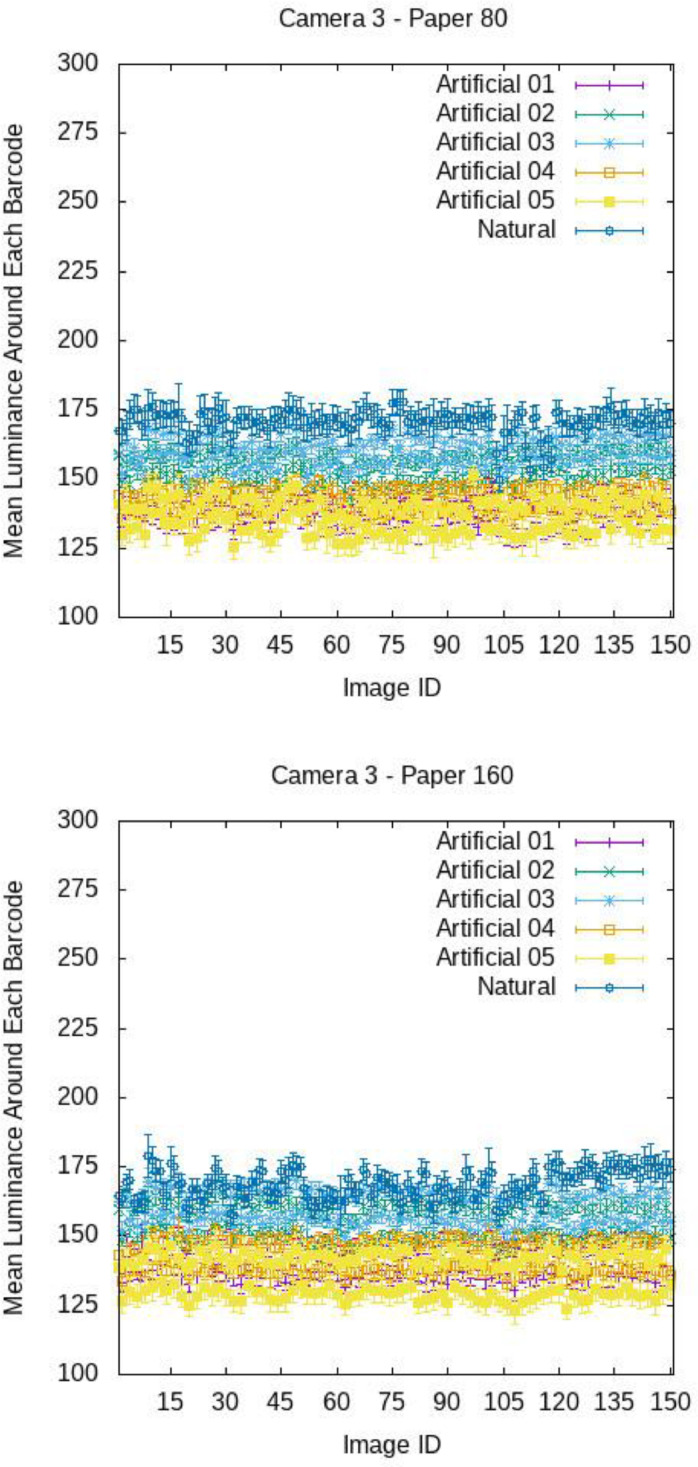
Mean luminance around each barcode for the various images of COLOUR-BARCODES-ON-WHITE-PAPER for Camera 3 and paper with density 80 gr/m^2^ (on top) and 160 gr/m^2^ (on bottom). X-axis reports the label of each image (i.e., 15 refers to the file 015.ppm).

Figs. 9, 10, and 11 report, for each image, the mean luminance U around each barcode with standard deviations, broken down by camera and paper. The x-axis reports the ID of the images, i.e., 15 stands for image 015.ppm. For each camera, paper and light, the plots exhibit an undulating trend. In fact, as already observed qualitatively, the brightness around on- top barcodes (see images with odd ID, e.g., 001.ppm, 003.ppm, …) is in generally higher than that around on-bottom barcodes (see images with even ID, e.g., 002.ppm, 004.ppm, …). Anyway, for each image, the standard deviation of U is small with respect to the mean value of U (see Tables 4 and 5 for a numerical comparison), meaning that, the light brightness can be considered uniform on the barcode regions.

**Table 4:** Mean values of the mean intensity and standard deviation of the region around each barcode in COLOUR-BARCODES for the acquisitions with paper with density 80gr/m^2^.

Light	Camera 1	Camera 2	Camera 3
Artificial-01	192 ± 2	171 ± 3	138 ± 3
Artificial-02	201 ± 2	179 ± 3	154 ± 3
Artificial-03	203 ± 2	177 ± 3	160 ± 3
Artificial-04	188 ± 2	168 ± 3	142 ± 3
Artificial-05	188 ± 2	170 ± 3	138 ± 5
Natural	203 ± 3	183 ± 5	171 ± 5

**Table 5:** Mean values of the mean intensity and standard deviation of the region around each barcode in COLOUR-BARCODES for the acquisitions with paper with density 160gr/m^2^.

Light	Camera 1	Camera 2	Camera 3
Artificial-01	177 ± 2	173 ± 3	140 ± 3
Artificial-02	195 ± 2	180 ± 3	155 ± 3
Artificial-03	192 ± 2	177 ± 3	161 ± 3
Artificial-04	177 ± 2	167 ± 3	143 ± 3
Artificial-05	187 ± 2	171 ± 3	136 ± 5
Natural	199 ± 3	190 ± 5	169 ± 4

**Fig. 12:**
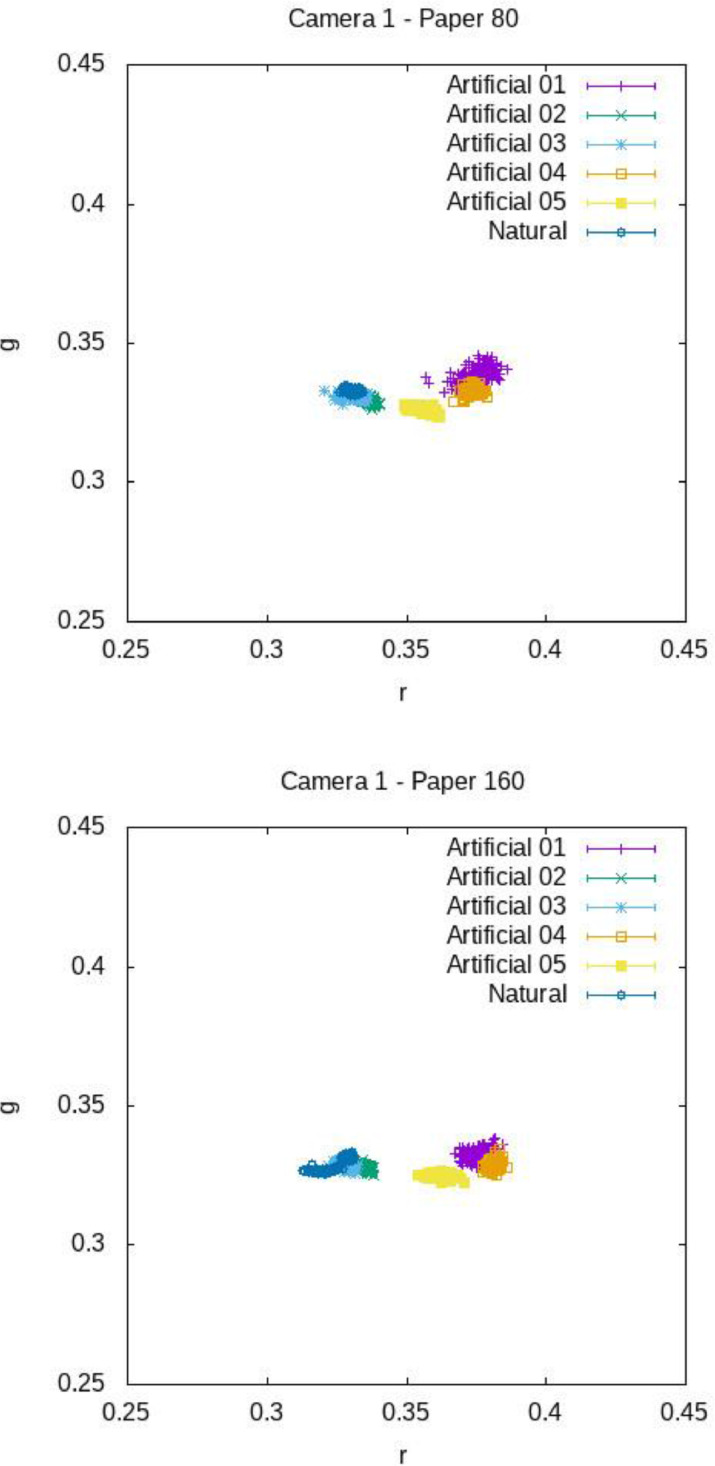
Mean chromaticity around each barcode for the various images of COLOUR-BARCODES-ON-WHITE-PAPER for Camera 1 and paper with density 80 gr/m^2^ (on top) and 160 gr/m^2^ (on bottom).

**Fig. 13:**
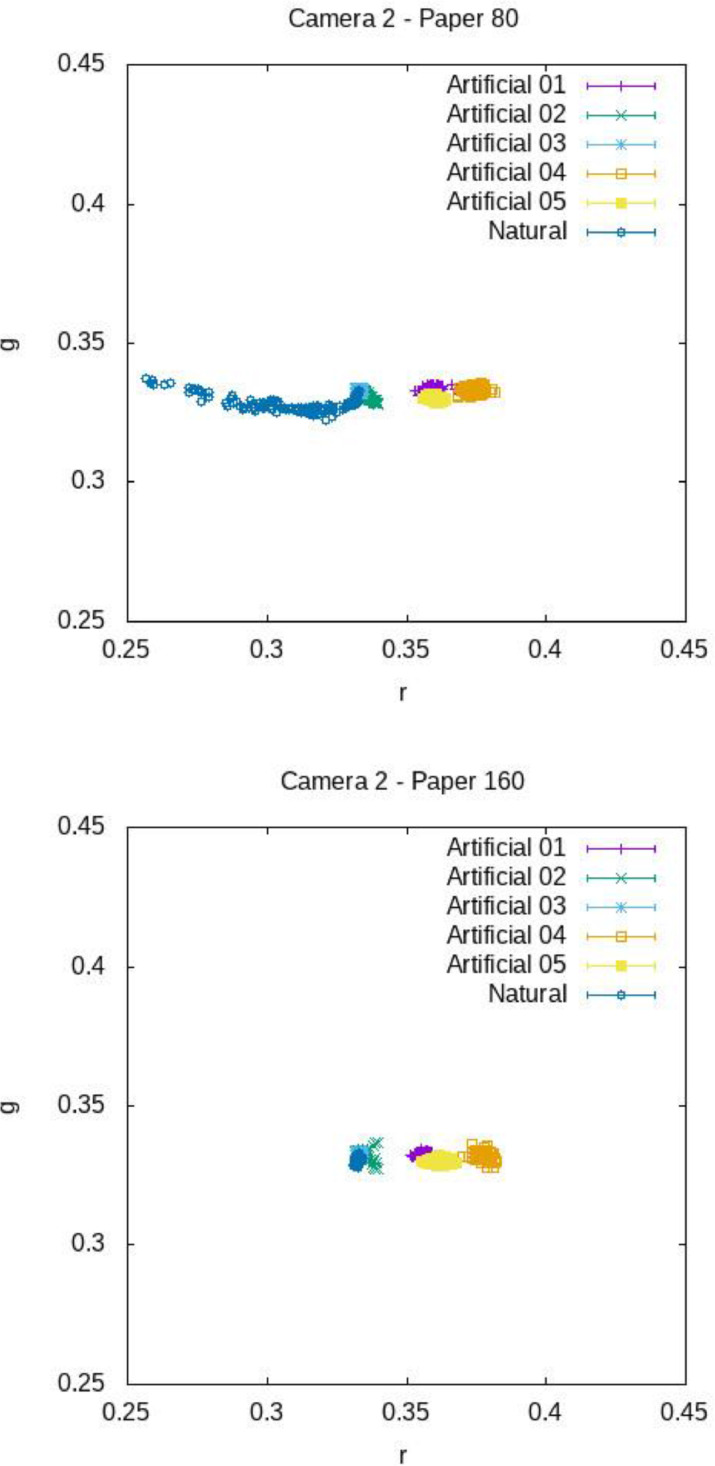
Mean chromaticity around each barcode for the various images of COLOUR-BARCODES-ON-WHITE-PAPER for Camera 2 and paper with density 80 gr/m^2^ (on top) and 160 gr/m^2^ (on bottom).

**Fig. 14:**
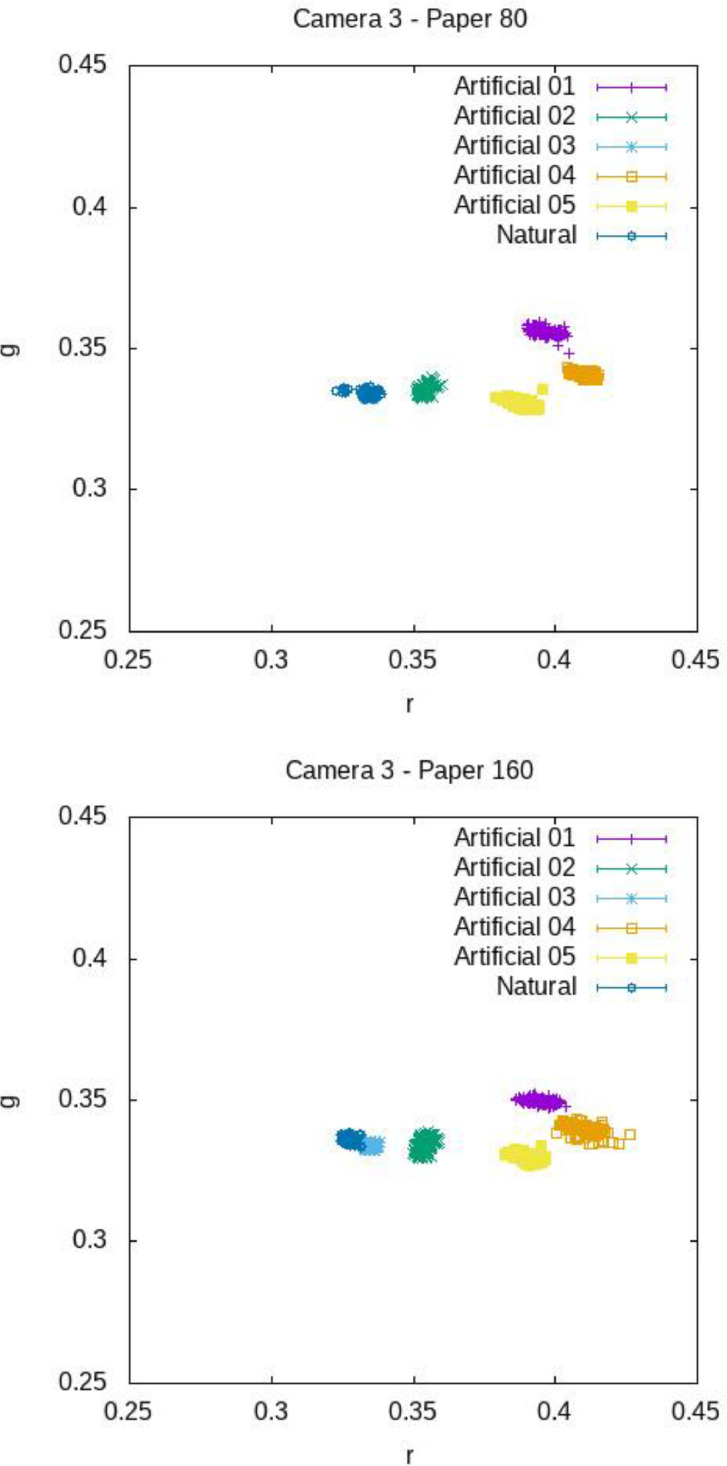
Mean chromaticity around each barcode for the various images of COLOUR-BARCODES-ON-WHITE-PAPER for Camera 3 and paper with density 80 gr/m^2^ (on top) and 160 gr/m^2^ (on bottom).

Fig. 12, Fig. 13, Fig. 14 show the mean chromaticity (r,g) around each barcode with standard deviation. Differently from U, there is no chromaticity gradient. The standard deviation of each chromaticity component c=r,g Is small with respect to the value of c (see Tables 6 and 7 for a numerical comparison), meaning that the light chromaticity can be considered stable over the barcode region and its surround.

**Table 6:** Mean values of the mean chromaticity (r and g) and standard deviation of the region around each barcode in COLOUR-BARCODES for the acquisitions with paper with density 80gr/m^2^.

Light	Component	Camera 1	Camera 2	Camera 3
Artificial-01	r	0.37477 ± 0.00322	0.35892 ± 0.00220	0.39685 ± 0.00396
	g	0.33834 ± 0.00215	0.33324 ± 0.00175	0.35581 ± 0.00263
Artificial-02	r	0.33496 ± 0.00236	0.33517 ± 0.00213	0.35391 ± 0.00299
	g	0.33014 ± 0.00180	0.33147 ± 0.00166	0.33519 ± 0.00209
Artificial-03	r	0.33181 ± 0.00217	0.33305 ± 0.00172	0.33401 ± 0.00257
	g	0.33111 ± 0.00167	0.33306 ± 0.00153	0.33385 ± 0.00204
Artificial-04	r	0.37401 ± 0.00277	0.37435 ± 0.00242	0.40950 ± 0.00363
	g	0.33289 ± 0.00186	0.33314 ± 0.00185	0.34090 ± 0.00227
Artificial-05	r	0.35573 ± 0.00319	0.35979 ± 0.00262	0.38767 ± 0.00519
	g	0.32601 ± 0.00186	0.33023 ± 0.00186	0.33065 ± 0.00285
Artificial-06	r	0.33059 ± 0.00187	0.31147 ± 0.00206	0.33401 ± 0.00303
	g	0.33311 ± 0.00138	0.32854 ± 0.00133	0.33400 ± 0.00215

**Table 7:** Mean values of the mean chromaticities and standard deviation of the region around each barcode in COLOUR-BARCODES for the acquisitions with paper with density 160gr/m^2^.

Light	Component	Camera 1	Camera 2	Camera 3
Artificial-1	r	0.37595 ± 0.00322	0.3559 ± 0.0022	0.39437 ± 0.00396
	g	0.33234 ± 0.00215	0.33223 ± 0.00175	0.34958 ± 0.00263
Artificial-2	r	0.3348 ± 0.00236	0.33433 ± 0.00213	0.35391 ± 0.00299
	g	0.32823 ± 0.0018	0.33243 ± 0.00166	0.33414 ± 0.00209
Artificial-3	r	0.32788 ± 0.00217	0.33315 ± 0.00172	0.33432 ± 0.00257
	g	0.32887 ± 0.00167	0.33286 ± 0.00153	0.33379 ± 0.00204
Artificial-4	r	0.38135 ± 0.00277	0.37743 ± 0.00242	0.40973 ± 0.00363
	g	0.32876 ± 0.00186	0.33224 ± 0.00185	0.33977 ± 0.00227
Artificial-5	r	0.36206 ± 0.00319	0.3613 ± 0.00262	0.38927 ± 0.00519
	g	0.32488 ± 0.00186	0.32982 ± 0.00186	0.32973 ± 0.00285
Natural	r	0.32593 ± 0.00187	0.33222 ± 0.00206	0.32807 ± 0.00303
	g	0.32964 ± 0.00138	0.33089 ± 0.00133	0.33607 ± 0.00215

For each camera, and for each light and paper, Table 4, Table 5 report the mean brightness of U , while Table 6, Table 7 report the mean value of (r,g). From these data, we can observe that Camera 3 reports warmer chromaticies and darker brightnesses than Camera 1 and Camera 2. Camera 1 and Camera 2 have a similar behaviour: for both these cameras, the chromaticities measured around the barcodes under Artificial-02 and Artificial-03 are very close to each other, although these lights have quite different correlated colour temperature. However, for Camera 2, U is lower than for than Camera 1 and the chromaticities of Artificial-01 and Artificial-05 are also very close to each other (see also Fig. 13).

Finally, we observe that, for the natural light, both chromaticiy and brightness change from image to image because this light depends on the time at which the acquisition is made (e.g., morning, afternoon, evening, …) as well as on other uncontrollable conditions, like sudden weather changes. This explains the remarkable variability of the values of (r,g) reported for the acquisitions made by Camera 2 and regarding the barcodes printed on paper with density 80gr/m^2^.

##### 3.2.3.2 Measuring Colour Barcode Size

Table 8 reports the mean value of the scale factor (with mean standard deviation) between the area of each barcode in the acquired images and the area of the corresponding barcode in COLOUR-BARCODES, averaged by the number of image acquisition (i.e., 150). The scale factor changes from camera to cameras because of the different cameras’ field of view, lens, and focus length. Slight changes of scale factors can be observed also from image to image of the same acquisition group (e.g., images captured by the same camera under a given illumination), because of the automatic adjustment of the camera settings and/or variation of the barcode sheet from the camera due to the manual positioning. Moreover, also printing process contributes to barcode rescaling. We finally point out that the size of the barcodes in the acquired images also depends on the barcode segmentation accuracy, thus the values in Table 8 refer to the masks we provided.

**Table 8:** Mean scale factor with standard deviation of the barcodes in the acquired images with respect to their size in COLOUR-BARCODES, broken down by paper density, cameras, and lights.

*Paper Density*	*Camera*	*Artificial-01*	*Artificial-02*	*Artificial-03*	*Artificial-04*	*Artificial-05*	*Natural*
*80* gr*/m^2^*	*1*	2.44 ± 0.15	2.61 ± 0.20	2.61 ±0.20	2.61 ± 0.20	2.62 ± 0.21	2.45 ± 0.15
	*2*	5.04 ± 0.39	4.97 ± 0.39	4.98 ± 0.40	4.95 ± 0.39	4.83 ± 0.38	4.95 ± 0.41
	*3*	3.61 ± 0.22	3.54 ± 0.22	3.52 ± 0.22	3.52 ± 0.23	3.91 ± 0.29	3.91 ± 0.33
*160* gr*/m^2^*	*1*	2.82 ± 0.18	2.39 ± 0.15	2.45 ± 0.15	2.45 ± 0.15	2.45 ± 0.15	2.60 ± 0.16
	*2*	5.18 ± 0.37	5.21 ± 0.33	5.22 ± 0.34	5.13 ± 0.33	5.14 ± 0.40	5.18 ± 0.35
	*3*	3.78 ± 0.24	3.69 ± 0.23	3.64 ± 0.23	3.62 ± 0.24	4.04 ± 0.28	3.40 ± 0.28

### 3.3 Images in COLOUR-BARCODES-ON-CLUTTERED-BACKGROUND

To enable testing algorithms for the extraction and decoding of colour barcodes in images with cluttered backgrounds, we collected 150 real-world pictures of indoor / outdoor environments, textured and uniform and objects like dolls, plants, food, and we saved them as JPG files in SYNTHETIC-COLOUR-BARCODES-ON-CLUTTERED-BACKGROUND (see Fig. 5). For each i = 1, …., 150 we superimposed on the i-th image the i-th colour barcode of COCO-10 by an algorithm that selected randomly the scale and the position of the barcode. Only in-plane rotation of 0, 90, 180, 270 degrees where allowed, while other angles were forbidden to avoid aliasing when printing the images.

We divided these 150 images into two groups (group 1 and group 2), each containing 75 images and we randomly assigned to each group a paper type: therefore, we printed the images of Group 1 on paper with density 160gr/m^2^, while the images of Group 2 on paper with density 80gr/m^2^.

In turn, we divided the printed images of each group G (*G* = 1, 2) into three subsets each containing 25 images and we acquired each of them under one of the six illuminants described above by two cameras.

In addition to Cameras 1, 2 and 3, here we considered three smartphone cameras (see Table 9). As for the acquisitions of the barcodes on white paper, automatic mode (in particular, automatic white balance) was always ON for all the cameras. The illuminations and the pairs of cameras used for the acquisitions were randomly assigned to the six image subgroups.

**Table 9:** Additional cameras used for acquiring the colour barcodes on cluttered backgrounds.

Label	Mark	Resolution
Camera 4	Xiaomi Redmi 9A [16]	3264×2448
Camera 5	Samsung Galaxy A14 [17]	4080×3060
Camera 6	LG-Bello II [18]	1920×2560

For these acquisitions, cameras were handled directly by authors (i.e., no tripods were used) with the recommendation to minimize blur, perspective skew and shadows on the barcode region. Due to this manual acquisition, barcodes sometimes appear at different scales and may be rotated by an arbitrary angle, usually different from the orientations of 0, 90, 180, 270 degrees used (see Fig. 5 for an example). We computed the mask of each image by manually selecting the barcode region and setting its pixels to zero, while the rest to one. In general, it was hard to exactly clip out the exact barcode region, and in fact the drawn rectangles are usually a bit larger than the region occupied by the barcode.

Table 10 reports the following information. The first column specifies which images belong to the first and second groups, precisely, group 1 (printed on the thicker paper) contains the printed versions of images img076.jpg, …, img150.jpg, while group 2 contains the printed versions of images img001.jpg, …, img075.jpg.

**Table 10:** This table reports for each group of printed images from COLOUR-BARCODES-ON-CLUTTERED-BACKGROUND the identifiers of the barcodes contained in each mage (second column), the light (third column) and the cameras (fourth column) used for the acquisition. The last column shows the mean scale factor (with standard deviation) at which barcodes are portrayed in the colour images.

Group	Identifiers of barcodes included in the images	Light	Cameras	Mean Scale Factor
0. (Paper with 160 gr/m^2^) Images img076.jpg, …, img150.jpg	from 76 to 100	Natural	1	5.40 ± 1.65
			4	7.70 ± 2.33
	from 101 to 125	Artificial-01	2	8.73 ± 3.25
			5	8.65 ± 3.22
	from 126 to 150	Artificial-03	3	5.19 ± 0.66
			6	4.75± 0.62
1. (Paper with 160 gr/m^2^) Images img001.jpg, …, img075.jpg	from 1 to 25	Artificial-04	1	5.36 ± 2.03
			4	7.22 ± 2.75
	from 26 to 50	Artificial-02	2	6.17 ± 2.12
			5	6.34 ± 2.21
	from 51 to 75	Artificial-05	3	5.59 ± 0.80
			6	5.42 ± 0.80

The second column report the identifiers of the barcodes contained in each image of the group in the first column. For instance, image img076.jpg depicts the barcode with identifier 76, i.e., the barcode saved as bc76.ppm in COLOUR-BARCODES, possibly rescaled and rotated by 0, 90, 180 or 270 degrees. The third and fourth columns show respectively the illumination and the cameras under which the images with the barcodes in the second column are acquired. For instance, the images img076.jpg, …, img100.jpg depicting the barcodes bc76.jpg, …, bc100.jpg, are captured under the light Natural by Cameras 1 and 4. Finally, the last column reports the mean value (with standard deviation) of the scale factor at which any barcode B is portrayed in the acquired image with respect to the image of B in COLOUR-BARCODES. In these images, the barcodes are much more enlarged than in the images of COLOUR-BARCODES-ON-WHITE-PAPER. The standard deviation values indicate that in the images with cluttered backgrounds, the barcode size has a higher variability than in the images in COLOUR-BARCODES-ON-WHITE-PAPER. These differences are due to the different distance at which the barcodes were taken, to the scale factor at which they appear on the synthetic images, as well as to the resizing made by the printers.

## Limitations

The dataset contains 5700 images of 150 color barcodes obtained using two types of paper, six cameras and six illuminations. Although the sample size can meet the pre-requisites of many applications, specially, in those making use of AI techniques, the database can be further extended by incorporating colors from a wider spectrum and paper types, such as glossy paper typical of the packaging of various commercial products.

Another addition to the present data may be the collection of images in less-than-optimal lighting conditions or from a paper medium that is not perfectly intact, as might occur in many practical contexts. This type of data containing a not-insignificant level of noise could in fact be used to make decoding algorithms even more efficient.

The dataset contains for now only barcode images, but it might prove very useful to add images of other information codes (e.g., QR codes) taken by the same methods.

## Ethics Statement

The authors have read and complied with the ethical requirements for publication in Data in Brief and confirm that the current work does not involve human subjects, animal experiments, or any data collected from social media platforms.

## CRediT authorship contribution statement

**Michela Lecca:** Conceptualization, Methodology, Software, Validation, Formal analysis, Investigation, Data curation, Writing – original draft, Writing – review & editing, Visualization, Supervision. **Paola Lecca:** Investigation, Resources, Writing – original draft, Writing – review & editing, Funding acquisition.

## Data Availability

COCO-10 (Original data) (Zenodo).
